# Mucinous nonneoplastic cyst of the pancreas penetrates the colon causing infection: a case report

**DOI:** 10.1186/s13256-019-2160-2

**Published:** 2019-08-10

**Authors:** Masashi Inoue, Ichiro Ohmori, Nozomi Karakuchi, Yuki Takemoto, Manabu Shimomura, Kazuaki Miyamoto, Masahiro Ikeda, Kazuhiro Toyota, Seiji Sadamoto, Tadateru Takahashi

**Affiliations:** Department of Surgery, National Hospital Organization Higashihiroshima Medical Center, 513 Jike, Saijo-cho, Higashihiroshima, Hiroshima 739-0041 Japan

**Keywords:** Pancreatic cyst, Mucinous nonneoplastic cyst (MNNC), Infection, Colon fistula

## Abstract

**Background:**

Mucinous nonneoplastic cyst of the pancreas is a rare disease defined as a cystic lesion lined with mucinous epithelium, supported by hypocellular stroma and not communicating with the pancreatic ducts. Mucinous nonneoplastic cyst of the pancreas has no malignant potential and does not require surgical resection or surveillance. However, its preoperative differentiation from other cystic lesions of the pancreas is difficult because of several overlapping clinical, radiological, and biochemical features. We report a rare case of large mucinous nonneoplastic cyst of the pancreas in which surgery was required due to infection and the possibility of malignancy.

**Case presentation:**

A 75-year-old Japanese man was found to have a pancreatic cyst in 2006 while undergoing postoperative evaluation for colon cancer. In 2015, the cyst ruptured, and it was treated conservatively. In 2017, he fell down on a road with a fever of 40 °C and was transported emergently to a nearby hospital. Enhanced computed tomography revealed a cystic lesion in the body of the pancreas measuring 119 mm × 100 mm and an adjacent left renal cyst measuring 63 mm in diameter. The wall of the pancreatic cyst was thickened. Magnetic resonance imaging demonstrated a liquid surface in the pancreatic cyst. Pancreatic cyst infection was diagnosed as the source of infection. However, identification of the organism was difficult. Furthermore, due to the increase in the size and wall thickness of the cyst, it was unclear whether the cystic mass was neoplastic with malignant potential. For these reasons, the patient underwent distal pancreatectomy and splenectomy with deroofing of the left renal cyst. Intraoperatively, the pancreatic cyst adhered to the descending colon, and partial resection of the colon was added. Pathologic analysis of the resected cyst demonstrated a simple cyst lined by mucinous epithelium. There was no underlying stromal condensation or epithelial dysplasia, and communication with the native pancreatic ducts was not observed. Based on the operative and histological findings, a final diagnosis of mucinous nonneoplastic cyst of the pancreas with colonic communication was made. The colonic fistula was presumed to be the source of infection.

**Conclusion:**

Mucinous nonneoplastic cyst of the pancreas is generally benign and requires little follow-up, but large cysts may penetrate other organs and cause severe complications.

## Introduction

Asymptomatic pancreatic cysts are being detected with increasing frequency due to widespread use of sensitive abdominal imaging tests such as multidetector computed tomography and magnetic resonance imaging (MRI). The classification of cystic pancreatic neoplasms is generally based on differentiating mucinous from nonmucinous cysts and benign from malignant cysts [1–3].

Mucinous nonneoplastic cyst of the pancreas (MNNC) is defined as a cystic lesion lined with mucinous epithelium, supported by hypocellular stroma and not communicating with the pancreatic ducts [4]. MNNC has no malignant potential and does not require surgical resection or surveillance. However, its preoperative differentiation from mucinous neoplasms includes mucinous cystic neoplasms (MCNs) or intraductal papillary mucinous cystic neoplasms (IPMNs), and it is difficult due to several overlapping clinical, radiological, and biochemical features [5, 6]. We report a case of MNNC in which surgery was required due to infection and the possibility of malignancy.

## Case presentation

**Fig. 1:**
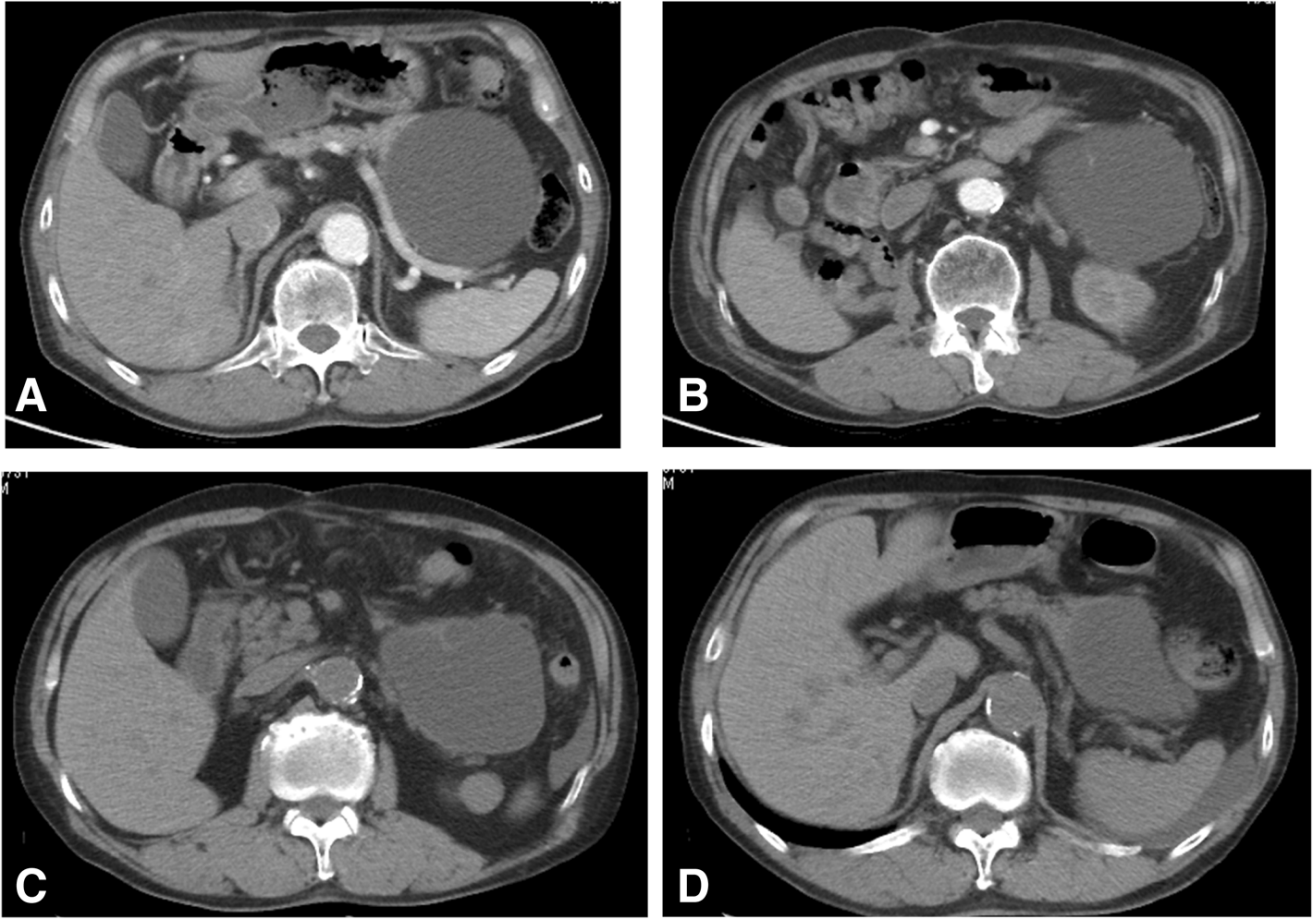
Prior computed tomography findings. In 2014, the pancreatic cyst size was 103 mm (**a, b**). In 2015, the pancreatic cyst ruptured, and the patient was treated conservatively (**c, d**)

A 75-year-old Japanese man was found to have a pancreatic cyst in 2006 while undergoing postoperative evaluation for colorectal cancer. The pancreatic cyst increased in size, and surgery was recommended, but the patient declined (Fig. 1a, b). In 2015, the cyst ruptured, and he was treated conservatively (Fig. 1c, d). In 2017, he began dialysis for chronic renal failure. The same year, he fell down on a road with a fever of 40 °C and was transported emergently to a nearby hospital. Laboratory tests showed elevated levels of C-reactive protein. Serum levels of carcinoembryonic antigen and carbohydrate antigen 19-9 were 5.8 ng/ml and 131.3 U/ml, respectively (Table 1).

**Table 1:** Blood test results at the time of admission

<complete blood count>			<biological examination>		
WBC	8300	/μl	Na	138	mEq/L
RBC	305×10^4^	/μl	K	3.1	mEq/L
Hgb	10.0	g/dl	Cl	101	mEq/L
Plt	42.0×10^4^	/μl	T-Bil	0.82	mg/dl
			AST	47	IU/l
<Tumor marker>			ALT	26	IU/l
CEA	5.8	ng/ml	BUN	58.1	mg/dl
CA19-9	131.3	U/ml	Cr	6.24	mg/dl
			LDH	312	IU/l
<Blood coagulation test>			ALP	240	IU/l
PT	99.8	%	γ-GTP	12	IU/l
APTT	27.9	sec	Amylase	27	IU/l
FDP	20.5	μg/ml	Glu	91	mg/dl
D-dimer	13.9	μg/ml			

*WBC* white blood cell, *RBC* red blood cell, *Hgb* hemoglobin, *Plt* platelet, *CEA* carcinoembryonic antigen, *CA19-9* carbohydrate antigen 19-9, *PT* prothrombin time, *APTT* activated partial thromboplastin time, *FDP* fibrin and fibrinogen degradation products, *Na* sodium, *K* potassium, *Cl* chlorine, *T-bil* total bilirubin, *AST* aspartate aminotransferase, *ALT* alanine aminotransferase, *BUN* blood urea nitrogen, *Cr* creatinine, *LDH* lactate dehydrogenase, *ALP* alkaline phosphatase, *γ-GTP* γ-glutamyltransferase, *Glu* glucose

**Fig. 2:**
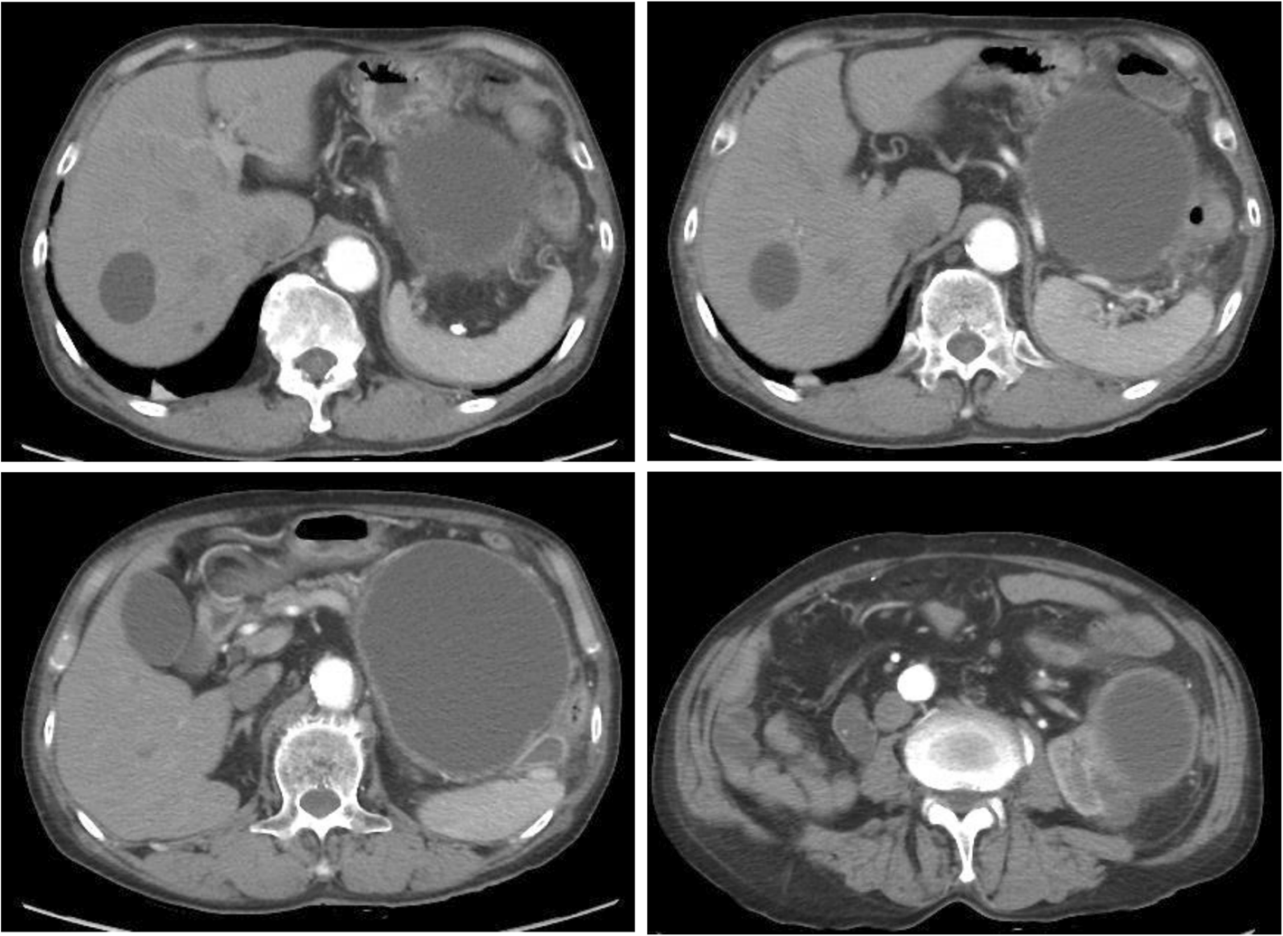
Computed tomography (CT) findings. Enhanced CT revealed a cystic lesion in the body of the pancreas measuring 119 mm × 100 mm and an adjacent left renal cyst of 63 mm in diameter. The pancreas cyst wall was thickened. The pancreatic ducts were not dilated

**Fig. 3:**
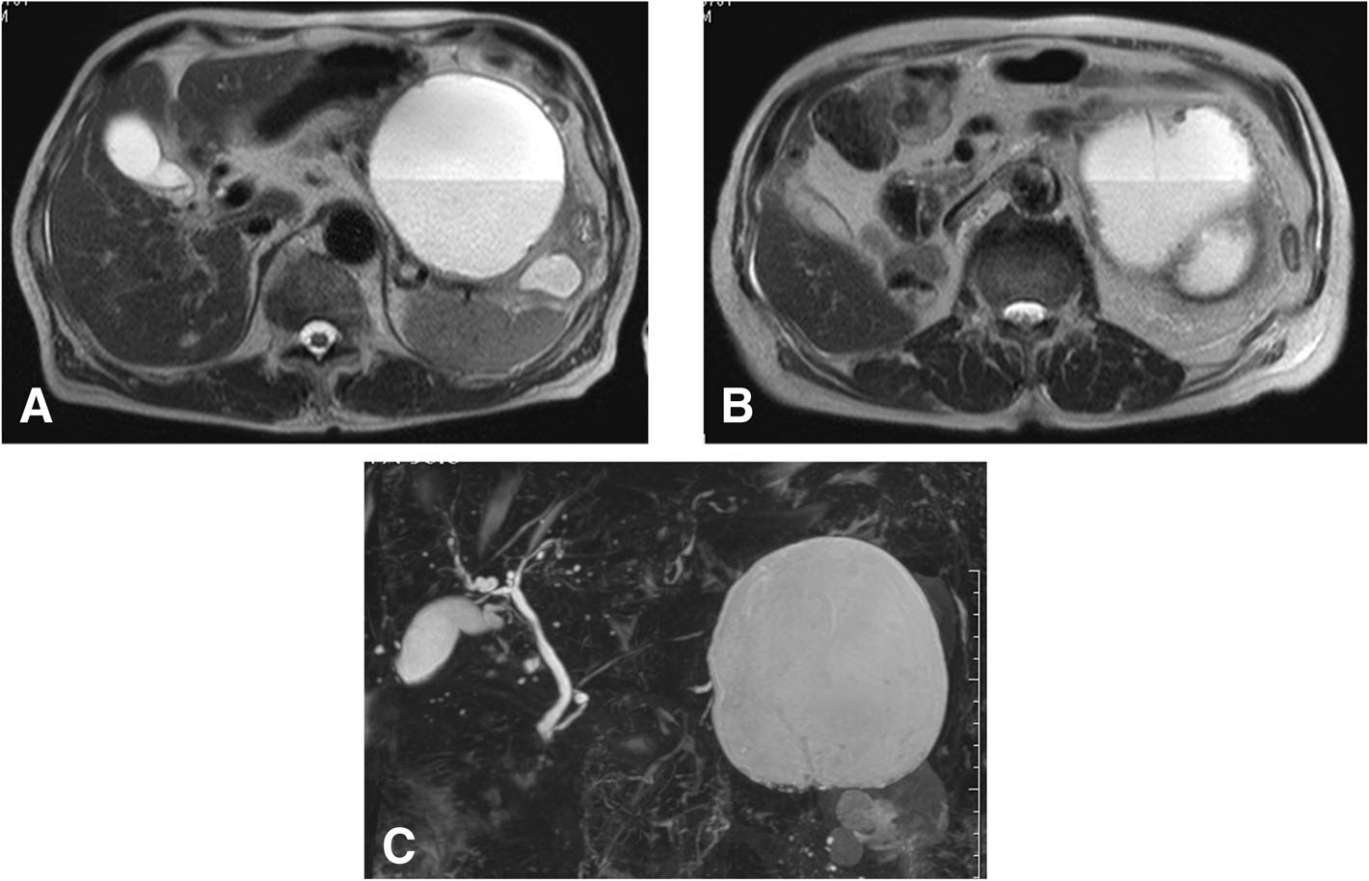
Magnetic resonance imaging (MRI) findings. MRI demonstrated a liquid surface in the pancreatic cyst (**a, b**). The cyst contained mucinous components suggesting infection. Magnetic resonance cholangiopancreatography did not show pancreatic duct dilation (**c**)

**Fig. 4:**
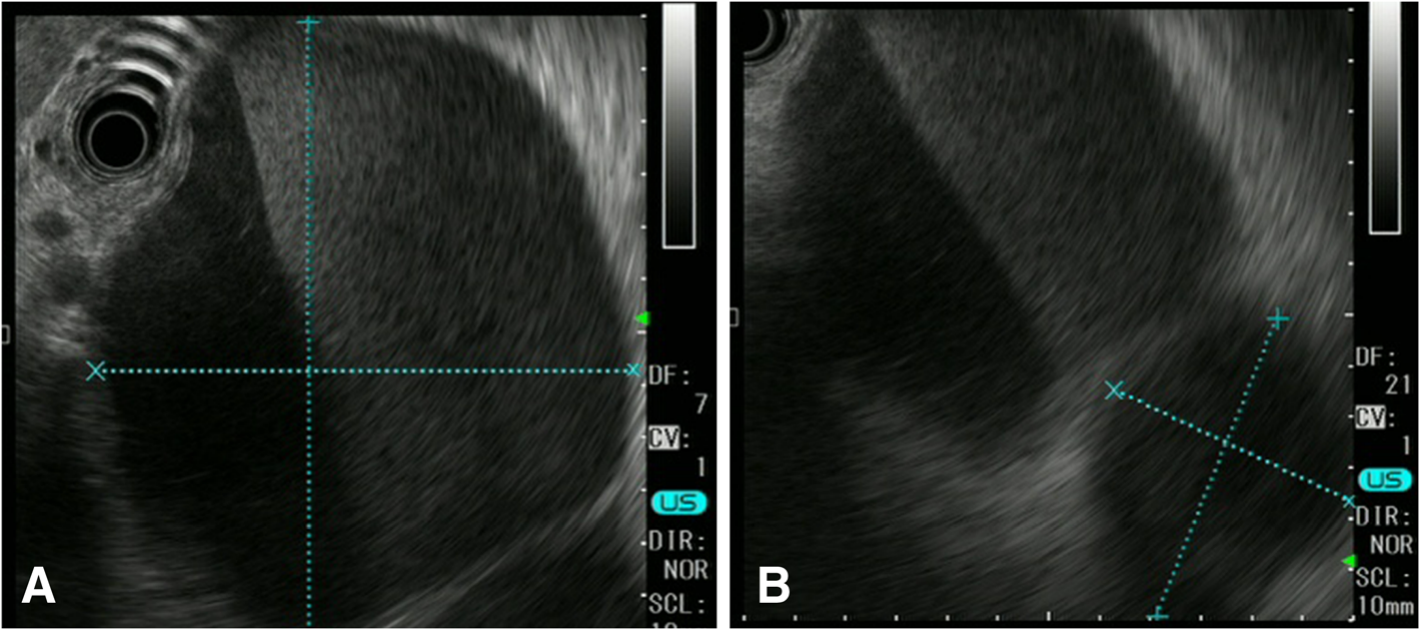
Endoscopic ultrasound (EUS) findings. **a** Pancreatic cyst. **b** Right renal cyst. EUS showed a liquid surface in the pancreatic cyst and did not show nodules in the cysts

**Fig. 5:**
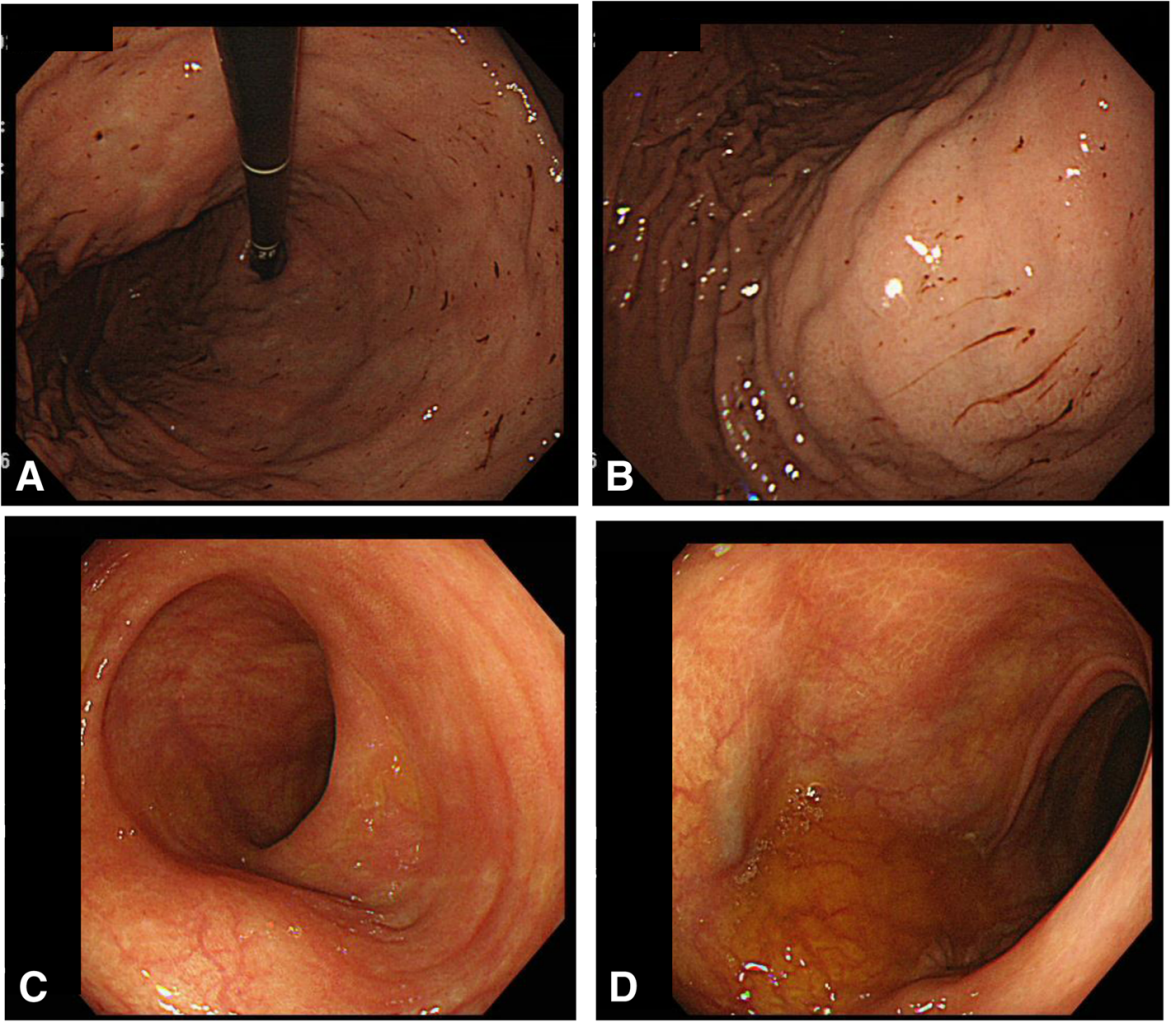
Endoscopy findings. Gastroscopy showed an elevated mass in the posterior wall of the body of the stomach with intact mucosa (**a, b**). Colonoscopy showed an elevated mass in the descending colon with intact mucosa (**c, d**)

The patient was treated with new quinolone-based antibiotics for 3 weeks as a conservative treatment after hospitalization. Bacteria were not detected by blood culture examination at admission. Enhanced computed tomography (CT) revealed a cystic lesion in the body of the pancreas measuring 119 mm × 100 mm and an adjacent left renal cyst measuring 63 mm in diameter. The wall of the pancreatic cyst was thickened. The pancreatic ducts were not dilated (Fig. 2). Magnetic resonance imaging (MRI) demonstrated a liquid surface in the pancreatic cyst (Fig. 3a, b). Magnetic resonance cholangiopancreatography (MRCP) did not show pancreatic duct dilation (Fig. 3c). Endoscopic ultrasound (EUS) showed a liquid surface in the pancreatic cyst and did not show nodules in the cyst (Fig. 4). Gastroscopy showed an elevated mass in the posterior wall of the body of the stomach with intact mucosa (Fig. 5a, b). In addition, colonoscopy performed 5 months before admission showed an elevated mass in the descending colon with intact mucosa (Fig. 5c, d).

Pancreatic cyst infection was diagnosed as the source of infection. However, identification of the organism was difficult. Furthermore, due to the increase in the size and wall thickness of the cyst, it was unclear whether the cystic mass was neoplastic with malignant potential. For these reasons, the patient underwent distal pancreatectomy and splenectomy, with deroofing of the left renal cyst. Intraoperatively, the pancreatic cyst strongly adhered to the descending colon, so partial resection of the colon was added.

**Fig. 6:**
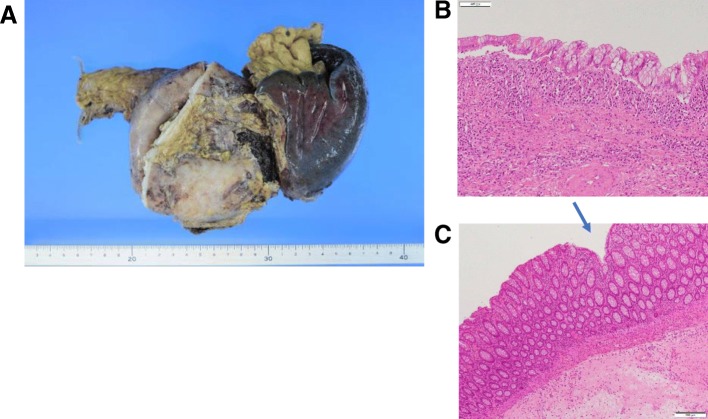
Pathologic analysis. **a** Macroscopic findings. **b** Microscopic findings of pancreas cyst wall. **c** Microscopic findings of colon mucosa. Pus accumulated inside the pancreatic cyst and renal cyst. The cyst was a simple cyst lined by mucinous epithelium. There was no underlying stromal condensation or epithelial dysplasia. Communication with the native pancreatic ducts was not observed. The resected colon adhesive pancreatic wall revealed a fistula-like depression on the mucosal side of the colon (↘).Mucinous nonneoplastic cyst of the pancreas fistula to the colon was the presumed source of infection

Pathologic analysis of the resected cyst demonstrated a simple cyst lined by mucinous epithelium (Fig. 6b). There was no underlying stromal condensation or epithelial dysplasia, and communication with the native pancreatic ducts was not observed. Pathological analysis of the resected colon adhesive pancreatic wall revealed a fistula-like depression on the mucosal side of the colon (Fig. 6c). *Bacteroides fragilis* and *Streptococcus sanguinis* were detected as a result of culture examination of the pancreatic cyst contents. On the basis of the operative and histological findings, a final diagnosis of MNNC with colon communication was made (Fig. 6). The small colonic fistula was presumed to be the source of infection.

Postoperatively, the patient was treated for a grade B pancreatic fistula. On postoperative day 12, emergency surgery was performed for thrombotic descending colon perforation. On postoperative day 94, the patient was discharged on foot.

## Discussion

We describe a case of a large MNNC that eroded into the colon, leading to cyst infection. MNNC is an increasingly recognized subset of pancreatic cysts. In 2002, Kosmahl *et al.* [7] reported a new type of nonneoplastic cystic lesion of the pancreas, designated as MNNC, in five patients. Later, 21 more cases were reported [8–11]. The incidence of MNNC has been reported as 2.1–3.4% in resected pancreatic lesions [8, 9]. MNNC has no malignant potential and does not require surgical resection or surveillance. MNNC is pathologically distinct from MCN in that it lacks an ovarian-type stroma, cellular atypia, and communication with the pancreatic ductal system distinctive of IPMN. Its preoperative differentiation from MCN or IPMN presents a diagnostic challenge due to several overlapping clinical, radiological, and biochemical features. Fisher *et al*. [12] studied the accuracy of CT in predicting the malignant potential of cystic pancreatic neoplasms in 48 patients. The authors found that CT diagnosis was accurate in 39% of cases. Visser *et al.* [13] reported on the relative accuracy of CT and MRI in the characterization of pancreatic cysts in 58 patients and showed that there was a substantial rate of misdiagnosis (54–57%). Another study compared the performance of CT and MRI/MRCP in the characterization of small pancreatic cysts in 30 patients [14]. MRI enabled more confident assessment of cyst morphology, but the accuracy of both imaging techniques for cyst characterization was comparable (71–84%). Diagnosis of cyst contents has been attempted, but it has not yet been reliably successful.

In our patient’s case, the cyst was previously identified on the basis of CT in 2006. Surgery was recommended at that time because the cyst was difficult to distinguish from a malignancy, but the patient refused. The cyst later ruptured and then developed a cryptic infection requiring extensive surgery.

We are aware of no prior case reports of MNNC and colonic fistula. However, severe acute pancreatitis can lead to localized complications such as the formation of a pseudocyst, necrosis, or an abscess. Acute pancreatitis complications with colonic involvement have a relatively low incidence of approximately 3% [15]. Fistula formation is thought to be due to elevated internal pressure from fluid accumulation within the pseudocyst and proteolytic enzymes within the fluid that invade adjacent organs, inducing ischemic changes that enable penetration of the walls of the most vulnerable organs and formation of a fistula [16]. Most pseudocyst enteric fistulas that form in the upper gastrointestinal tract can be treated conservatively and have a relatively good prognosis. In contrast, fistulas that form in the colon rarely heal spontaneously and tend to be associated with fatal complications, with a reported mortality of approximately 17–67% [17].

In our patient’s case, it was presumed that the increased MNNCs were in communication with the colon, resulting in a small colon fistula through which enteric bacteria could pass, causing a cystic infection. CT revealed increased pancreatic cyst size and wall thickness. MRI suggested a mucinous component of the cyst. It was difficult to evaluate the whole cyst in detail by EUS. For these reasons, we were unable to rule out malignancy and chose to proceed with surgical resection. Surgical intervention allows for complete removal of the cyst and provides tissue for an accurate histological diagnosis.

Because the cyst communicated with the descending colon, a partial resection of the colon was also performed. It is possible that ischemic changes caused by the resection led to the subsequent colonic rupture. The colon rupture was unpredictable as a postoperative complication, but it should be considered when similar cases are seen in the future.

## Conclusion

This was a case of a large MCCN that caused a fistula to the descending colon, through which the cyst became infected. MCCN is generally benign and requires little follow-up, but large cysts may penetrate other organs and cause severe complications.

## Data Availability

The datasets generated during the current study available from the corresponding author on reasonable request.

## Abbreviations

CT: Computed tomography
EUS: Endoscopic ultrasound
IPMN: Intraductal papillary mucinous cystic neoplasm
MCN: Mucinous cystic neoplasm
MNNC: Mucinous nonneoplastic cyst of the pancreas
MRCP: Magnetic resonance cholangiopancreatography
MRI: Magnetic resonance imaging
